# Niclosamide Triggers Non-Canonical LC3 Lipidation

**DOI:** 10.3390/cells8030248

**Published:** 2019-03-15

**Authors:** Yajun Liu, Xia Luo, Hao Shan, Yuanyuan Fu, Qianqian Gu, Xueping Zheng, Qi Dai, Fan Xia, Zhihua Zheng, Peiqing Liu, Xiao-Ming Yin, Liang Hong, Min Li

**Affiliations:** 1 School of Pharmaceutical Sciences, Guangdong Provincial Key Laboratory of New Drug Design and Evaluation, National and Local United Engineering Lab of Druggability and New Drugs Evaluation, Sun Yat-Sen University, Guangzhou, Guangdong 510006, China; liuyajun915@gmail.com (Y.L.); luoxia3706@163.com (X.L.); 17827063336@163.com (H.S.); fuyy6@mail2.sysu.edu.cn (Y.F.); guqq@mail2.sysu.edu.cn (Q.G.); jnzxp1993@126.com (X.Z.); dqdq0908@126.com (Q.D.); xiaf6@mail2.sysu.edu.cn (F.X.); zhengzhihualllleng@163.com (Z.Z.); liupq@mail.sysu.edu.cn (P.L.); 2 Department of Pathology and Laboratory Medicine, Indiana University School of Medicine, Indianapolis, IN 46202, USA; xmyin@iu.edu

**Keywords:** autophagy, bafilomycin A1, Golgi complex, niclosamide, non-canonical LC3 lipidation, vimentin

## Abstract

Autophagy is a highly- evolutionarily-conserved catabolic pathway activated by various cellular stresses. Recently, non-canonical autophagy (NCA), which does not require all of the ATG proteins to form autophagosome or autophagosome-like structures, has been found in various conditions. Moreover, mounting evidence has indicated that non-canonical LC3 lipidation (NCLL) may reflect NCA. We and others have reported that niclosamide (Nic), an anti-helminthic drug approved by the Food and Drug Administration, could induce canonical autophagy via a feedback downregulation of mTOR complex 1. In this study, we found that Nic could also induce NCLL, which is independent of the ULK1 complex and Beclin 1 complex, but dependent on ubiquitin-like conjugation systems. Although bafilomycin A1 and concanamycin A, two known V-ATPase inhibitors, significantly inhibited Nic-induced NCLL, Nic-induced NCLL was demonstrated to be independent of V-ATPase. In addition, the Golgi complex and vimentin were involved in Nic-induced NCLL, which might be a platform or membrane source for Nic-induced LC3-positive structures. These results would be helpful to broaden our understanding of the working mechanisms of Nic and evaluate its pharmacological activities in diseases.

## 1. Introduction

Drug discovery and development are costly and lengthy processes. Therefore, drug repurposing has attracted increasing attention in recent years [1]. Drug repurposing means finding novel functions for existing drugs, which is more economical and faster than the canonical drug development strategy, as new uses of old drugs that have been well characterized and approved for human use could be evaluated rapidly in clinical trials [2].

Niclosamide (Nic), a salicylanilide derivative, is on the World Health Organization’s list of essential medicines and has been approved by the Food and Drug Administration (FDA) as an anti-helminthic drug. Considerable studies have suggested that Nic is a multifunctional drug and has the potential to be a candidate for the treatment of various diseases [3]. One of its biological activities is to induce autophagy. We and others have demonstrated that Nic could induce canonical autophagy via a feedback downregulation of mTOR complex 1 [4,5,6]. Autophagy, a conserved lysosomal catabolic process in eukaryotic cells for degrading and recycling intracellular components, is essential for cellular homeostasis [7]. Autophagy is active under normal circumstances and enhanced by several stress situations, such as nutrient deprivation, hypoxia, and oxidative stress. During the past two decades, great breakthroughs have been made in the molecular mechanisms of autophagy. This dynamic pathway is modulated by a set of autophagy-related (ATG) proteins that comprise several functional complexes, including the ULK1 (unc-51-like autophagy activating kinase 1) complex (consisting of ULK1, FIP200, and ATG13), the Beclin 1 complex (consisting of Beclin 1, class III phosphatidylinositol-3 kinase (PI3KC3), and ATG14), the ATG12-ATG5-ATG16L1 complex (consisting of ATG12, ATG5, and ATG16L1), and ATG8/LC3, to form the autophagosome [8]. In general, autophagy could be divided into three stages: initiation and nucleation, elongation and closure, fusion and degradation [9]. Each stage forms a membrane structure: phagophore, autophagosome, and autolysosome, respectively. Upon stimulation, the ULK1 complex and Beclin 1 complex are activated to form the phagophore and generate PI3P for promoting the recruitment of the ATG12-ATG5-ATG16L1 complex and ATG8/LC3. These two ubiquitin-like conjugation systems are crucial for the elongation and closure of the autophagosomal membrane [10]. Finally, the closed autophagosome fuses with the lysosome to form the autolysosome and degrade cargo. These procedures and involved ATG proteins are essential for canonical autophagy.

Mounting evidence has indicated that mutations or polymorphisms of *ATG* genes are associated with various human diseases, such as cancer, neurodegenerative diseases, and infectious diseases [11,12]. In addition, several FDA-approved drugs, including rapamycin, metformin, and clonidine, and commonly-used nutritional supplements, including vitamin D, caffeine, and spermidine, have been confirmed to induce autophagy and may exert their clinical efficacy via autophagy [13]. According to these progressions, autophagy has been considered as a potential therapeutic target for diverse diseases, and great interest has been sparked to find potent modulators of autophagy. However, it is possible that the mutations or polymorphisms of *ATG* genes in diseases, which may result in autophagy deficiency, would also block the upregulation of autophagy by compounds. Therefore, it is more practical to screen compounds that could induce autophagy in the absence of some *ATG* genes.

Recently, non-canonical autophagy (NCA), which does not require all of the ATG proteins to form autophagosome or autophagosome-like structures, including single-membrane and double-membrane structures, has been found in various conditions. However, our understanding of NCA is still in its childhood. We do not know whether NCA has some special functions, and the relationship between NCA and canonical autophagy is still unclear. Generally, NCA relies on only a portion of ATG proteins to achieve the same function as canonical autophagy in sequestering intracellular components or invading pathogens and ultimately degrading in the lysosomal compartment [14]. Thus, NCA may possess a wider application prospect. Screening and developing NCA modulators could be a new direction for various disease therapies. Therefore, it is necessary to explore their working mechanisms in NCA.

Beclin 1-indepent autophagy was the first-described NCA [15]. It could be triggered by many proapoptotic chemicals including MK801, gossypol, and Z18 [16,17,18]. Subsequently, it was reported that ULK1-independent NCA could be induced by ammonia and glucose deprivation [19]. In most of these treatments, double-membrane autophagosomes, which are traditionally defined as the signature in canonical autophagy, have also been detected. However, with the recent progress in the studies of NCA, there is more and more evidence showing that the membrane structures of NCA are not strictly limited to double-membraned vesicles. LC3-assosiated phagocytosis (LAP), a non-canonical autophagic pathway to eliminate pathogens or dead cells, has demonstrated that the recruitment of LC3 to the phagosome, a single-membrane structure, is independent of the ULK1 complex, but dependent on the ATG12-ATG5-ATG16L1 complex [20], and it is also defined as a type of NCA. In addition, except for ATG5/ATG7-independent autophagy [21], the LC3 lipidation assay was the common method for evaluating the level of NCA in these studies. Thus, NCA is in general dependent on ubiquitin-like conjugation systems. LC3 could also be an appropriate marker for the majority of NCA, and non-canonical LC3 lipidation (NCLL) may reflect NCA.

In this study, we are the first to discover that Nic could initiate NCLL, indicating that Nic may also induce NCA. This non-canonical process required integration of the Golgi complex and involved vimentin. We also found that bafilomycin A1 (Baf) could inhibit Nic-induced NCLL at an early stage in a V-ATPase- and Ca^2+^-independent manner. Determination of the precise mechanisms of NCLL induced by Nic from different angles could be helpful to evaluate its multiple pharmacological activities.

## 2. Materials and Methods

### 2.1. Chemicals and Antibodies

Nic (481909) and BAPTA (196419) were from Calbiochem (San Diego, CA, USA). Baf (B-1080) was from LC Laboratories (Woburn, MA, USA). 3-Methyladenine (3MA, S2767), brefeldin A (BFA, S7046), and golgicide A (GCA, S7266) were from Selleckchem (Houston, TX, USA). Ammonia chloride (AC, 191406) was from MPbio (Santa Ana, CA, USA). Thapsigargin (TG, MB13319) and ionomycin (Iono, MB7511) were from Meilunbio (Dalian, China).

Antibodies to MAP1LC3B (PM036), ATG5 (M153), and ATG16L1 (M150) were from MBL International (Woburn, MA, USA); antibodies to ATG12 (2011) and vimentin (5741) were from Cell Signaling Technology (Danvers, MA, USA); antibodies to LC3B (L7543), α-tubulin (T6074), and β-actin (A1978) were from Sigma (St. Louis, MO, USA); antibody to WIPI2 (ab105459) was from Abcam (Cambridge, UK); antibody to GM130 (610822) was from BD Bioscience (San Jose, CA, USA). Secondary antibodies conjugated with Alex Fluor 488, Alex Fluor 594, or horseradish peroxidase were from Invitrogen and Jackson Immuno Research Laboratories, respectively.

### 2.2. Cell Culture and Transfection

Wild-type Hela cells, *Beclin 1*-knockdown U251 cells, wild-type mouse embryo fibroblast cells (MEFs), *FIP200*-, *ULK1*-, and *ATG5*-deficient MEFs, and *ATG4B*-deficient Hela cells have been described previously [22,23]. Cells were cultured in DMEM (Thermo Scientific, SH3024301, Rockford, IL, USA) supplemented with 10% (*v*/*v*) fetal bovine serum (Gibco, A31608, Grand Island, NY, USA) and standard supplements in a 37 °C, 5% (*v*/*v*) CO_2_ incubator. For starvation, Earle’s balanced salt solution (EBSS, Sigma, E2888, St.Louis, MO, USA) was used. Cells were grown in 12-well plates before transfection, and siRNA (GenePharma, Shanghai, China) was transfected into cells using Lipofectamine 2000 (Invitrogen, Carlsbad, CA, USA) for gene knockdown.

### 2.3. Immunoblot Assay

For the immunoblot assay, 20–30 μg of whole cell lysates were prepared for Western blot analysis. Samples were run on 10% or 12% SDS-PAGE gels and transferred to PVDF membranes (Millipore). After blocking and incubation with primary and secondary antibodies, specific proteins were detected using enhanced chemiluminescence Western blot agent (Millipore, Billerica, MA, USA). Images were taken using Kodak Image Station 4000 (Carestream Health, Rochester, NY, USA) or ImageQuant LAS 4000mini (GE, Uppsala, Sweden) with the companion software.

### 2.4. Immunostaining Assay

Cells were grown on 96-well plates and fixed in 4% paraformaldehyde for 15 min. Cells were washed twice in phosphate-buffered saline (PBS) followed by permeabilization with 0.1% Triton X-100 for 15 min and blocked in PBS containing 5% bovine serum albumin for 1 h. Primary antibodies were used at 4 °C overnight, followed by secondary antibodies for 1 h at room temperature. Fluorescence images were taken using EVOS FL Auto (Life Technologies, Bothell, WA, USA). For manual quantification of the puncta formation, at least 3 optical fields with at least 50 cells per experimental condition were analyzed. Data from repeated experiments were subjected to statistical analysis.

### 2.5. Molecule Capture

To measure the interaction between immobilized Nic and flowing cell lysates, the PlexArray HT System (Plexera LLC, Woodinville, WA, USA) was used to monitor the whole procedure in real time. The optical architecture and operation details of the PlexArray HT System were as described previously [24]. Cell lysates were prepared in phosphate-buffered saline with Tween-20 (PBST, pH 7.4), and glycine-HCl (pH 2.0) was used as the regeneration buffer. A typical binding curve was obtained by flowing the cell lysate sample at 2 μL/s for 300 s of association and then using PBST to wash the biochip 3 times at 2 μL/s for 300 s. Then, we used trypsin (Promega, Madison, WI, USA) to digest the proteins binding on the chip at 37 °C for 12 h. The peptide was separated by the Nano Acquity UPLC System (Waters, Milford, MA, USA) and identified by Q Exactive (Thermo Scientific, Waltham, MA, USA).

### 2.6. Statistical Analysis

The results shown are representative of at least three independent experiments. The data are presented as the mean ± standard deviation. Statistical significance was assessed by Student’s *t*-test. *p* < 0.05 was considered significant.

## 3. Results

### 3.1. Nic-Induced NCLL Is Independent of ULK1 Complex and Beclin 1 Complex, But Dependent on Ubiquitin-Like Conjugation Systems

**Figure 1:**
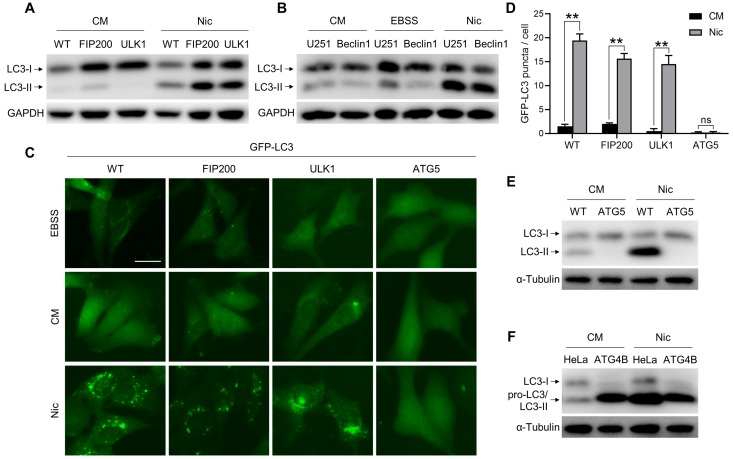
Nic could initiate non-canonical LC3 lipidation (NCLL), which is independent of the ULK1 complex and Beclin 1 complex, but dependent on the ubiquitin-like conjugation systems. (**A**) Wild-type MEFs (WT), *FIP200*KO-MEFs (FIP), and *ULK1*KO-MEFs (ULK) were treated with Nic (10 μM) for 6 h, and then, LC3-II formation was analyzed by immunoblotting. (**B**) Wild-type U251 cells (U251) and *Beclin 1*KD-U251 cells (Beclin 1) were treated with Nic (10 μM) for 6 h or EBSS for 2 h, then LC3-II formation was analyzed by immunoblotting. (**C**) WT-MEFs, *FIP200*KO-MEFs, *ULK1*KO-MEFs, and *ATG5*KO-MEFs stably expressing GFP-LC3 were treated with Nic (2.5 μM) for 6 h, and then, the GFP-LC3 puncta formation was analyzed. (**D**) Quantification of GFP-LC3 puncta in (C). (**E**) WT-MEFs and *ATG5*KO-MEFs were treated with Nic (10 μM) for 6 h, and then, the LC3-II formation was evaluated. (**F**) WT-HeLa cells (HeLa) and *ATG4B*KO-HeLa cells (ATG4B) were treated with Nic (10 μM) for 6 h and then analyzed by immunoblotting. CM, complete medium. ** *p* < 0.005. ns, not significant. Bar = 25 μM.

The ULK1 complex and Beclin 1 complex are essential for the early stage of autophagy. Unexpectedly, we found that Nic could still trigger LC3 lipidation in *FIP200*KO-MEFs, *ULK1*KO-MEFs, and *Beclin 1*KD-U251, cells as well as in wild-type MEFs (Figure 1A,B and Supplementary Figure S1G), whereas EBSS-induced canonical LC3 lipidation was abolished in these knockout/knockdown cells (Figure 1B and Supplementary Figure S1E,F). Consistent with the LC3-II formation, the number of GFP-LC3-positive structures was significantly increased with Nic treatment (Figure 1C,D). However, the non-canonical LC3 lipidation and GFP-LC3 puncta formation induced by Nic were abolished in *ATG5*KO-MEFs (Figure 1C–E). Moreover, we checked the role of ATG4B in Nic-initiated NCLL. In canonical autophagy, ATG4B is responsible for the LC3 conversion. In line with *ATG5*KO-MEFs, there was no LC3-II formation following Nic treatment in *ATG4B*KO-HeLa cells (Figure 1F). Taken together, these data suggested that Nic could induce NCLL. In Nic-induced NCLL, the ULK1 complex and Beclin 1 complex were dispensable, whereas the ubiquitin-like conjugation systems were imperative.

### 3.2. Nic Recruits the ATG12-ATG5-ATG16L1 Complex via a WIPI2-Independent Pathway

**Figure 2:**
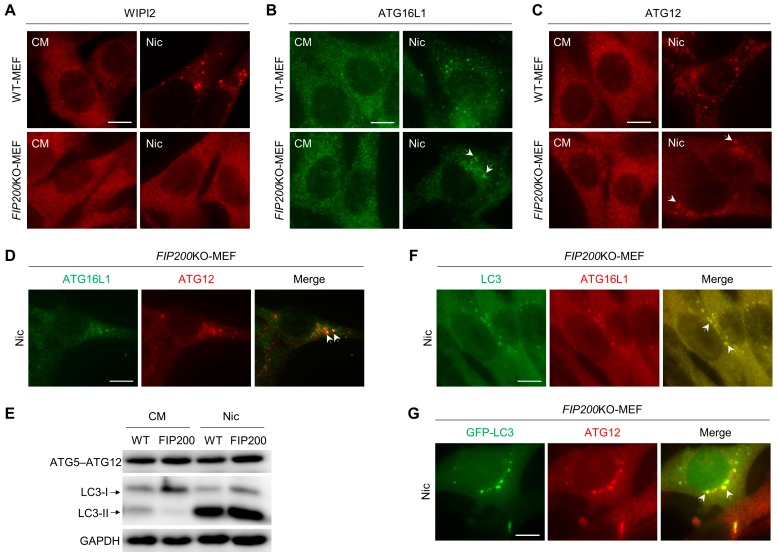
Nic-induced NCLL recruits ATG16L1 in a WIPI2-independent manner. (**A**–**C**) WT-MEFs and *FIP200*KO-MEFs were treated with Nic (10 μM) for 6 h, and then, the WIPI2 (**A**), ATG16L1 (**B**), and ATG12 (**C**) puncta formations were assessed by immunostaining. (**D**,**E**) *FIP200*KO-MEFs were treated with Nic (10 μM) for 6 h, and then, the colocalization of ATG16L1 and ATG12 (D) and the ATG5-ATG12 conjugation (E) were analyzed. (**F**) *FIP200*KO MEFs were treated with Nic (2.5 μM) for 6 h, followed by immunostaining of ATG16L1 and LC3. (**G**) *FIP200*KO-MEFs stably-expressing GFP-LC3 were treated with Nic (2.5 μM) for 6 h, followed by immunostaining of ATG12. CM, complete medium. Bar = 10 μM.

In canonical autophagy, PI3P, a product of PI3KC3, is required for autophagosome biogenesis and maturation [25]. The generation of PI3P on the phagophore facilitates the recruitment of two ubiquitin-like conjugation systems. It has been reported that this process is meditated by WIPI2, a PI3P effector protein. WIPI2 could directly bind to ATG16L1 and act immediately upstream of ATG16L1 [26]. Meanwhile, ATG16L1 binds to ATG15-ATG12 to form the ATG12-ATG5-ATG16L1 complex, which serves as an E3-like enzyme to generate LC3-II. Consequently, we evaluated the formation of WIPI2, ATG16L1, and ATG12 puncta after Nic stimuli. In wild-type MEFs, WIPI2, ATG16L1, and ATG12 puncta were all induced by Nic, but only ATG16L1 and ATG12 dots were detected in *FIP200*KO-MEFs (Figure 2A–C and Supplementary Figure S2A–C). Subsequently, we analyzed the colocalization of ATG12 and ATG16L1 puncta and the conjugation of ATG5 and ATG12. ATG12- and ATG16L1-positive structures were almost completely colocalized (Figure 2D and Supplementary Figure S2D), and ATG5-ATG12 conjugation was unaffected (Figure 2E) with Nic treatment. Moreover, we found that Nic-induced ATG16L1- and ATG12-positive puncta were also colocalized with Nic-induced LC3-positive structures (Figure 2F,G and Supplementary Figure S2E,F). These data indicated that the recruitment of ATG12-ATG5-ATG16L1 complex induced by Nic was independent of WIPI2.

### 3.3. Baf Is Able to Inhibit Nic-Induced NCLL 

**Figure 3:**
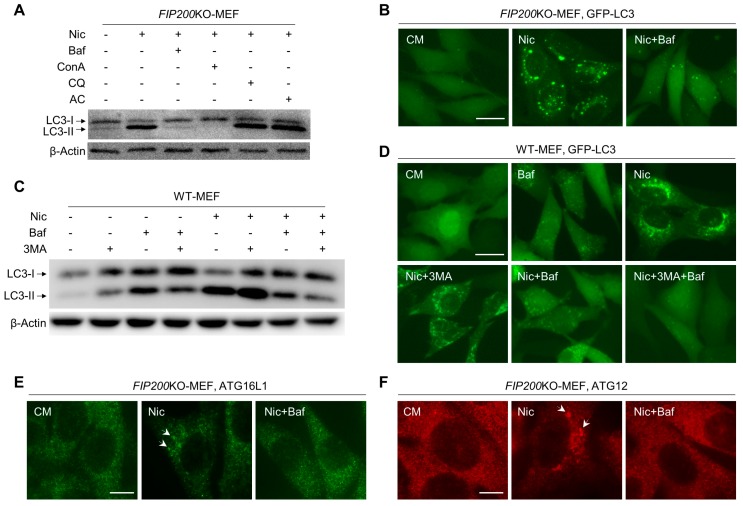
Nic-induced NCLL could be inhibited by Baf. (**A**) *FIP200*KO-MEFs were treated with Nic (10 μM) in the presence or absence of Baf (0.5 μM), concanamycin (ConA) (1 μM), chloroquine (CQ) (40 μM), or ammonia chloride (AC) (20 mM) for 6 h, and then, the LC3-II formation was analyzed. (**B**) *FIP200*KO-MEFs were treated by Nic (10 μM) with or without Baf (0.5 μM) for 6 h, and then, the GFP-LC3 puncta formation was assessed. (**C**) WT-MEFs were treated by Nic (10 μM) with or without 3MA (10 mM) or Baf (0.5 μM) for 6 h, and then, the LC3-II formation was assessed. (**D**) WT-MEFs were treated with Nic (10 μM) in the presence or absence of Baf (0.5 μM) and/or 3MA (10 mM) for 6 h, and then, the GFP-LC3 puncta formation was assessed. (**E**,**F**) *FIP200*KO-MEFs were treated by Nic (10 μM) with or without Baf (0.5 μM) for 6 h, and then, the ATG16L1 (**E**) and ATG12 (**F**) puncta formations were assessed.CM, complete medium. Bar = 10 μM.

Bafilomycin A1 (Baf), chloroquine (CQ), and ammonia chloride (AC) have been commonly used as inhibitors of the late stage of canonical autophagy [27]. However, we found that only Baf could inhibit the Nic-induced LC3-II formation and GFP-LC3 punctation in *FIP200*KO-MEFs, whereas CQ and AC had no extra effect on Nic-activated NCLL (Figure 3A,B). Similar to Baf, concanamycin (ConA), another well-known vacuolar-type H^+^-ATPase (V-ATPase) inhibitor, could also attenuate Nic-induced LC3 lipidation (Figure 3A). In wild-type MEFs, Baf alone could already decrease Nic-induced LC3-II formation; while both Baf and 3MA were present, LC3 lipidation was almost completely abolished (Figure 3C,D and Supplementary Figure S3A,B). This suggested that NCLL, which was inhibited by Baf, and canonical autophagy, which was blocked by 3MA, coexisted in WT-MEFs with Nic treatment. In addition, in *FIP200*KO-MEFs, Baf could eliminate the ATG16L1 and ATG12 puncta triggered by Nic without altering the expression level of ATG16L1 and ATG5-ATG12 conjugation (Figure 3E,F and Supplementary Figure S3C), indicating that Baf might directly interfere with ATG16L1 or its upstream signal. Taken together, Baf could be an early phase inhibitor of Nic-induced NCLL.

### 3.4. Nic-Induced NCLL Is Independent of V-ATPase and Ca^2+^

**Figure 4:**
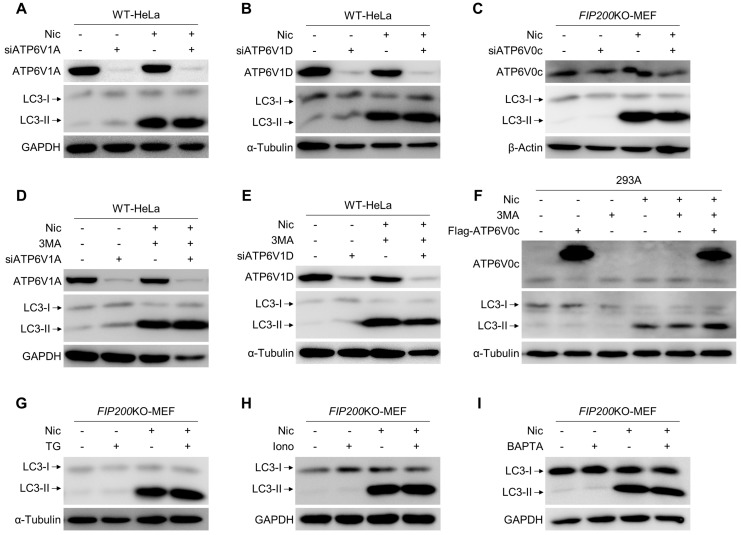
Nic-induced NCLL is independent of V-ATPase and Ca^2+^. (**A**,**B**) The siRNA of *ATP6V1A* (siATP6V1A) or *ATP6V1D* (siATP6V1D) was transfected to WT-HeLa cells, followed by treatment with Nic (10 μM) for 6 h, and then, the LC3-II formation was analyzed. © The siRNA of *ATP6V0c* (siATP6V0c) was transfected to *FIP200*KO-MEFs, followed by treatment with Nic (10 μM) for 6 h, and then, the LC3-II formation was evaluated. (**D**,**E**) The siRNA of *ATP6V1A* (siATP6V1A) or *ATP6V1D* (siATP6V1D) was transfected to WT-HeLa cells, followed by treatment with Nic (10 μM) in the presence or absence of 3MA (10 mM) for 6 h, and then, the LC3-II formation was analyzed. (**C**,**F**) The Flag-ATP6V0c plasmid was transfected to 293A cells, followed by treatment with Nic (10 μM) in the presence or absence of 3MA (10 mM) for 6 h, and then, the LC3-II formation was analyzed. (**G**–**I**) *FIP200*KO-MEF were treated by Nic with or without TG (3 μM), ionomycin (Iono, 2 μM), or BAPTA (10 μM) for 6 h, and then, LC3-II formation was analyzed by immunoblotting.

We have shown that Baf and ConA, two V-ATPase inhibitors, could block Nic-induced NCLL. V-ATPase consists of two domains, V0 and V1, and contains at least 13 subunits. To investigate the role of V-ATPase in NCLL, we knocked down three subunits of V-ATPase, *ATP6V1A*, *ATP6V1D*, and *ATP6V0c*, respectively. However, in HeLa cells, Nic-induced LC3-II formation was unaffected by the knockdown of *ATP6V1A* or *ATP6V1D* (Figure 4A,B), even in the presence of 3MA (Figure 4D,E). ATP6V0c was the reported target of Baf and ConA, but neither knocking it down, nor overexpressing it could change the LC3-II level upregulated by Nic (Figure 4C,F). These results indicated that V-ATPase might not be required for Nic-induced NCLL.

Interestingly, Baf has also been observed to be a moderately good inhibitor of P-ATPase [28], and SERCA, a type of P-ATPase, shows an intermediate sensitivity to Baf [29]. Thapsigargin (TG), a plant-derived sesquiterpene lactone, has been identified as a specific inhibitor of SERCA [30]. It was used widely as an ER stressor to induce autophagy, but recently shown in fact to block autophagy [31]. However, we found that TG could not inhibit Nic-triggered NCLL (Figure 4G). Subsequently, since both V-ATPase and SERCA have effects on the uptake and/or efflux of Ca^2+^, we applied two chemicals to change the intracellular calcium concentration to investigate the role of Ca^2+^ in Nic-induced NCLL. The Western blot results showed that both ionomycin, an ionophore that could raise the intracellular level of calcium, and BAPTA, a chelator that could reduce the intracellular level of calcium, were not able to block Nic-induced non-canonical LC3-II formation (Figure 4H,I). These data demonstrated that Ca^2+^ was dispensable for Nic to trigger NCLL.

### 3.5. Nic-Induced NCLL Requires Intact Golgi Complex

**Figure 5:**
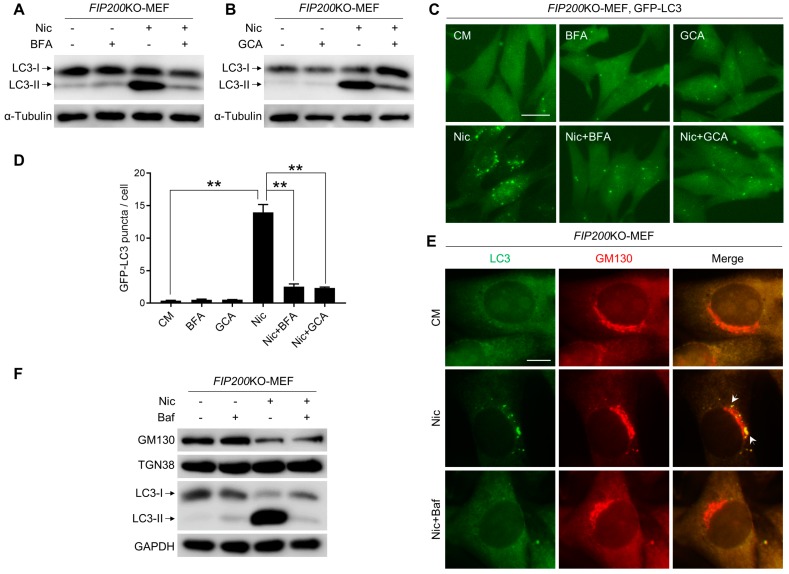
Nic-induced NCLL requires intact Golgi complex. (**A**,**B**) *FIP200*KO-MEFs were treated by Nic (10 μM) with BFA (5 μg/mL) (A) and GCA (20 μM) (**B**) for 6 h, and then, the LC3-II formation was analyzed. (**C**) *FIP200*KO-MEFs expressing GFP-LC3 were treated by Nic (2.5 μM) with BFA (5 μg/mL) and GCA (20 μM) for 6 h, and then, the GFP-LC3 puncta formation was assessed. (**D**) Quantification of GFP-LC3 puncta in (C). (**E**) *FIP200*KO-MEFs were treated with Nic (2.5 μM) for 6 h, followed by immunostaining of GM130 and LC3. (**F**) *FIP200*KO-MEFs were treated with Nic (10 μM) for 6 h, and then, the expression levels of GM130 and TGN38 were analyzed. CM, complete medium. ** *p* < 0.005. Bar = 10 μM.

It has been reported that the Golgi complex is vital for several types of NCA [23,32]. To confirm whether the Golgi apparatus is required for Nic-induced NCLL, brefeldin A (BFA) and golgicide A (GCA), two well-studied Golgi toxins that cause Golgi complex collapse, were used. Although both BFA and GCA were demonstrated to trigger canonical autophagy, they were all able to reduce the formation of LC3-II and GFP-LC3 puncta induced by Nic (Figure 5A–D). Furthermore, Nic-induced LC3 puncta overlapped with the *cis*-Golgi complex marker, GM130 (Figure 5E). Unexpectedly, the expression level of GM130 was significantly decreased by Nic, while TGN38 was not affected, and Baf could not reverse it (Figure 5F). These results suggested that integrated Golgi was required for Nic-induced NCLL, and the Golgi apparatus, especially the *cis*-Golgi complex, may contribute to autophagosome formation following Nic treatment.

### 3.6. Vimentin Is Involved in Nic-Induced NCLL

**Figure 6:**
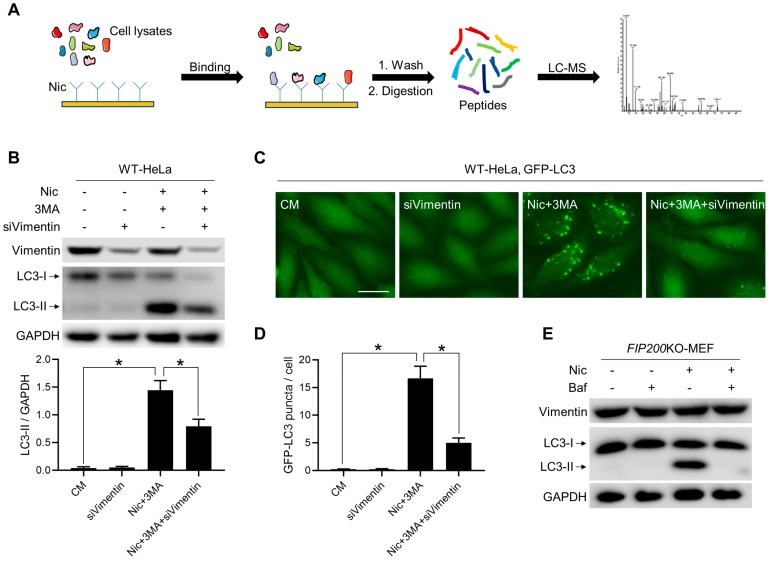
Vimentin is involved in Nic-induced NCLL. (**A**) The working procedure of “molecule capture”. (**B**) The siRNA of *vimentin* (siVimentin) was transfected to WT-HeLa cells, followed by treatment with Nic (10 μM) in the presence or absence of 3MA (10 mM) for 6 h, and then, the LC3-II formation was analyzed. (**C**) WT-HeLa cells expressing GFP-LC3 were transfected with siVimentin, followed by treatment with Nic (10 μM) in the presence or absence of 3MA (10 mM) for 6 h, then the GFP-LC3 puncta formation was assessed. (**D**) Quantification of GFP-LC3 puncta in (C). (**E**) *FIP200*KO-MEFs were treated with Nic (10 μM) with or without Baf (0.5 μM) for 6 h, and then, the expression level of vimentin was analyzed. * *p* < 0.05. Bar = 25 μM.

Recently, CQ, a conventional inhibitor of autophagy flux, was found to induce non-canonical LC3 lipidation [33], and this process is regulated by osmotic imbalances. Since both Nic and CQ could induce canonical autophagy through mTORC1 suppression and were all able to induce NCLL, we also explored the role of osmotic imbalances and water flux in the non-canonical LC3 lipidation triggered by Nic. Two mechanistically-distinct aquaporin inhibitors, phloretin and mercury chloride, were used to inhibit water influx. It was observed that both phloretin and mercury chloride could partially inhibit NCLL induced by Nic (Supplementary Figure S4), but the inhibitory effect was not as significant as Baf. In order to find a more precise target protein, we utilized “molecule capture” and got more than 90 protein candidates (Figure 6A and Supplementary Table S1). Among these candidates, vimentin got the highest score. Currently it is the only one we have found that could influence Nic-induced NCLL. We found that knocking *vimentin* down could significantly inhibit Nic-induced NCLL (Figure 6B–D), as well as AMDE1- and CCCP-induced NCLL, two known NCLL [23,34] (Supplementary Figure S5B,C), while the mRNA level of LC3B was not affected by knocking *vimentin* down (Supplementary Figure S5A). In addition, neither Nic nor Baf could influence the expression level of vimentin (Figure 6E), indicating that the interaction between vimentin and other proteins may play a critical role in Nic-induced NCLL. Otherwise, since vimentin is a structural protein, it is also possible that vimentin acts as a platform for recruiting NCLL-required proteins.

## 4. Discussion

Nic is an FDA-approved anti-helminthic drug. It has been used in millions of people to treat tapeworm infection for nearly 40 years, whereas its mechanism of action has not been well elucidated. Mounting evidence has demonstrated that Nic modulates diverse signaling pathways and biological processes, including uncoupling of oxidative phosphorylation, autophagy, and the Wnt/β-catenin signaling pathway [3], indicating that beyond application in parasitic infection treatment, it can also be exploited in other diseases. Here, we show a new function of Nic to help enlarge its application fields.

Autophagy dysfunction has been linked to a wide range of human diseases, and considerable enthusiasm has emerged to discover and develop autophagy inducers for the prevention and treatment of diseases [13]. However, this treatment strategy has certain limitations. The mutations or polymorphisms of *ATG* genes would possibly suppress the autophagy induced by candidate compounds. Therefore, NCA may possess a much wider prospect of application as a treatment target than canonical autophagy. An interesting point is how to find novel modulators of NCA. Although LC3 lipidation is not observed in ATG5/ATG7-independent alternative autophagy, in most types of NCA, functional autophagosomes or autophagosome-like structures are still decorated by LC3. Thus, NCA might represent LC3-positive structures and could be detected by the LC3 lipidation assay. In this study, we found that Nic might initiate NCA via evaluating the expression level of LC3-II and the number of GFP-LC3 puncta.

The ULK1 complex and Beclin 1 complex are involved in the initiation of canonical autophagy. However, in the absence of ULK1 or Beclin 1, Nic could still induce LC3 lipidation (Figure 1A–C), indicating that Nic-induced NCLL could be initiated in a ULK1- and Beclin 1-independent manner. These observations suggested that Nic could be used to treat diseases when the canonical initiation systems are compromised. Moreover, considerable enthusiasm has emerged for using Nic to treat cancer [3]. It has been demonstrated that Nic could treat more than 11 types of cancers in cell and animal models. Currently, on ClinicalTrial.gov, four clinical trials of Nic are recruiting participants for prostate cancer and colorectal cancer treatment. It is known that Beclin 1-independent NCA is induced by many proapoptotic compounds [14]. Nic is also able to trigger apoptosis, and a recent study reported that Nic-induced Wnt signaling inhibition in colorectal cancer is mediated by autophagy [6]; thus, there is a possibility that Nic-induced Beclin 1-independent NCA is involved in the therapy process and results in an autophagic cell death.

In canonical autophagy, PI3P, a product of PI3KC3, is required for autophagosome biogenesis [25]. However, recently, VPS34-independent NCA has been reported. In *VPS34^−/−^* T lymphocytes and sensory neurons, LC3 lipidation and autophagosomes have been observed [35,36]. However, WIPI2 puncta, an effector of PI3P, was only found in WT-MEFs, but not observed in *FIP200*KO-MEFs with Nic treatment (Figure 2A and Supplementary Figure S2A). Thus, PI3P may not be required for Nic-induced NCLL. The inhibitory effect of 3MA (Figure 3C,D and Supplementary Figure S3A,B) was due to its function in Nic-induced canonical autophagy, as canonical autophagy and NCLL coexisted in WT-MEF with Nic treatment.

ATG16L1 has several functional domains that play an essential role in autophagy. The N-terminal of ATG16L1 is responsible for interacting with ATG5-ATG12 conjugation to form the ATG12-ATG5-ATG16L1 complex, which performs as an E3-like enzyme to generate LC3-II [37]. The central region of ATG16L1 could bind to FIP200 and WIPI2, and this region is indispensable for starvation-activated canonical autophagy, but is not required for glucose deprivation-induced ULK1-independent NCA [26,38]. The C-terminal WD40 domain of ATG16L1 was recently reported to be used to distinguish canonical and non-canonical autophagy [39]. In this study, although we did not investigate which domain of ATG16L1 is required for Nic-induced NCLL, we found that in the absence of FIP200, Nic could also recruit ATG16L1 via a WIPI2-independent way (Figure 2A,B and Supplementary Figure S2A,B). In line with the previous study, we also found that Baf, a V-ATPase inhibitor, could abolish the ATG16L1 dots’ formation and LC3 lipidation triggered by Nic (Figure 3E), suggesting Baf could inhibit Nic-induced NCLL at the early stage. Thus, we predicted that V-ATPase activity should be required for Nic-initiated non-canonical LC3 lipidation. However, neither knockdown of *ATP6V1A*, *ATP6V1D*, or *ATP6V0c*, nor overexpression of *ATP6V0c* could inhibit Nic-induced NCLL (Figure 4A–F), indicating that V-ATPase may not be required, and the exact targets of Baf need further studies.

Various membrane structures, the Golgi complex, ER, mitochondria, and plasma membrane, could contribute to autophagosomes’ biogenesis, and among these, the Golgi complex may serve as a common platform for canonical autophagy and NCA [40]. It has been reported that Beclin 1-independent NCA induced by *αSNAP* depletion is associated with Golgi complex fragmentation [41]. In contrast, disrupting the Golgi complex by BFA or GCA could suppress *cis*-unsaturated fatty acid-induced Beclin 1-independent NCA [32]. Here, we demonstrated that BFA and GCA also inhibited Nic-triggered NCLL (Figure 5A–D). Furthermore, we observed that Nic-induced LC3 puncta overlapped with the *cis*-Golgi marker, GM130 (Figure 5E), indicating that the Golgi complex could potentially be the source of the membrane for Nic-induced LC3-positive structures. Meanwhile, we found that Nic could decrease the expression level of GM130, and there were some Golgi fragments after Nic treatment (Figure 5E,F). However, disruption of Golgi architecture and function has been widely observed in neurodegenerative diseases and the deletion of GM130 would result in cell death [42], which suggest that Nic might be toxic for neurons and may not be a good choice for neurodegenerative disease treatment.

## 5. Conclusions

In summary, we have provided evidence of Nic-induced ULK1- and Beclin 1-independent, but conjugation system-dependent NCLL, which could be significantly inhibited by Baf at the early stage. In addition, an intact Golgi complex and vimentin were involved in Nic-induced NCLL, which might be a membrane source or a platform for Nic-induced LC3-positive structures. Moreover, we have also provided several mechanistic views on how Nic could modulate NCLL, which would be helpful for an in-depth exploration of the pharmacological actions of Nic.

## Supplementary Materials

The following are available online at https://www.mdpi.com/2073-4409/8/3/248/s1, Figures S1–S5, Table S1.
