# Supplementary material for: Niclosamide Triggers Non-Canonical LC3 Lipidation
Source: Cells. 2019 Mar 15;8(3):248. doi: 10.3390/cells8030248 (PMC6468753; doi:10.3390/cells8030248)
Supplement: Supplementary file 1 [file cells-08-00248-s001.pdf]

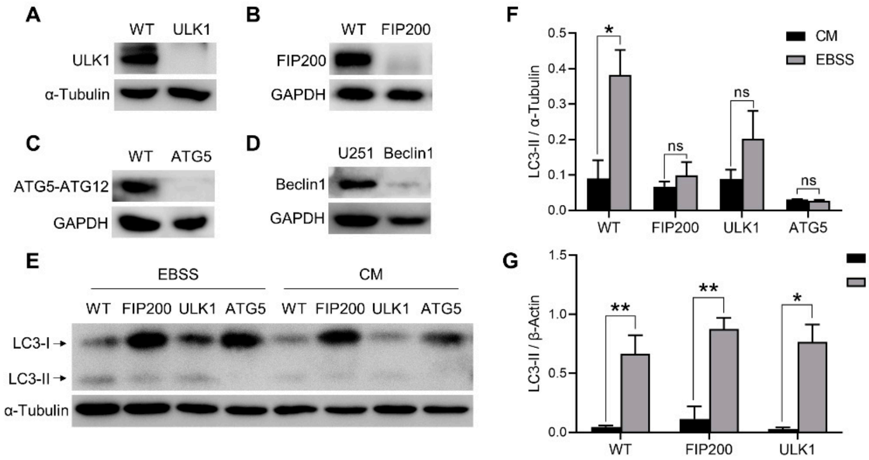

Figure S1. Validation of *ULK1*KO-, *FIP200*KO- and *ATG5*KO-MEFs, and *Beclin1*KD-U251 cells. (A-D) The expression levels of ULK1, FIP200, ATG5 and Beclin1 were detected in *ULK1*KO-MEFs (ULK1), *FIP200*KO-MEFs (FIP200), *ATG5*KO-MEFs (ATG5), and *Beclin1*KD-U251 cells (Beclin1), respectively. (E) Wild-type MEFs (WT), *FIP200*KO-MEFs (FIP200), *ULK1*KO-MEFs (ULK1) and *ATG5*KO-MEFs (ATG5) were treated with EBSS for 2h, then analyzed by immunoblotting. (F) Quantification of LC3-II/α-Tubulin in (E). (G) Quantification of LC3-II/β-Actin in Figure 1A. CM, complete medium. *p<0.05, **p<0.005. ns, not significant.

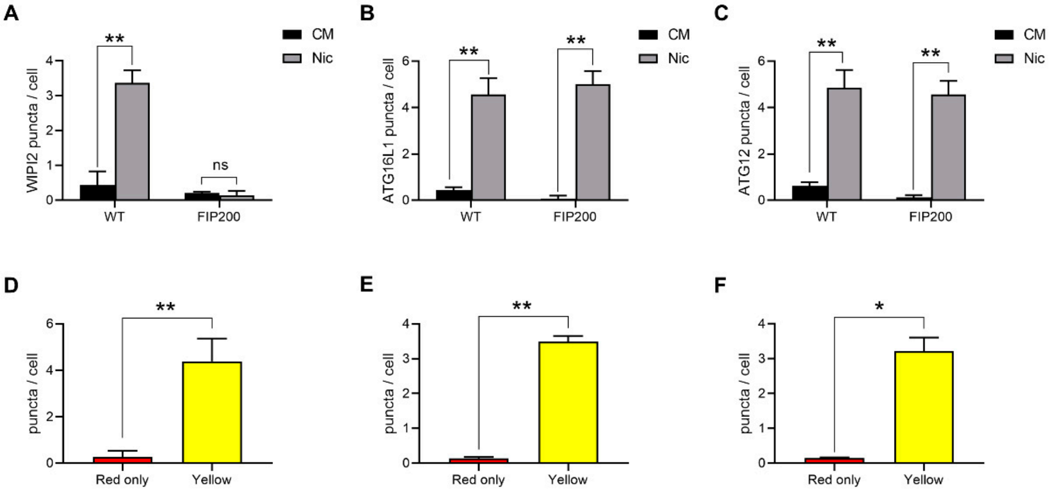

Figure S2. Quantification of puncta in Figure 2. (A-D) Quantification of WIPI2 (A), ATG16L1 (B), and ATG12 (C) puncta, and the colocalization of ATG16L1 and ATG12 puncta in Figure 2A-D. (E-F) Quantification the colocalization of ATG16L1 and LC3 (E) and ATG12 and LC3 (F) in Figure 2F and G. CM, complete medium. *p<0.05, **p<0.005. ns, not significant.

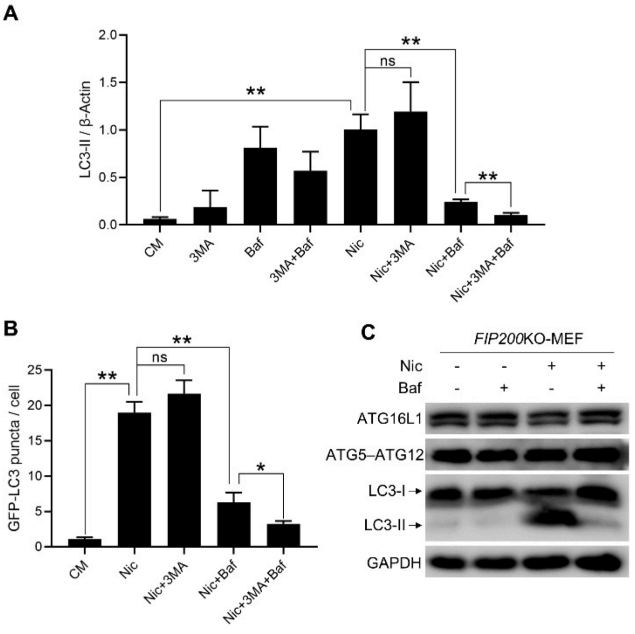

Figure S3. Nic-induced NCLL could be inhibited by Baf. (A) Quantification of LC3-II/β-Actin ratio in Figure 3C. (B) Quantification of GFP-LC3 puncta in Figure 3D. (C) *FIP200*KO-MEFs were treated by Nic (10 μM) with or without Baf (0.5 μM) for 6 h and then the expression levels of ATG16L1 and ATG5-ATG12 were analyzed. CM, complete medium. **p*<0.05, ***p*<0.005. ns, not significant.

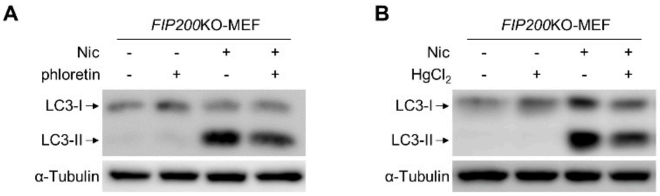

Figure S4. Nic-induced NCLL was regulated by osmotic imbalances. (A-B) *FIP*200KO-MEFs were treated with Nic (10 μM) in the presence or absence of phloretin (200 μM) or mercury chloride ($HgCl_2$, 2 μM) for 6 h and then the LC3-II formation was analyzed.

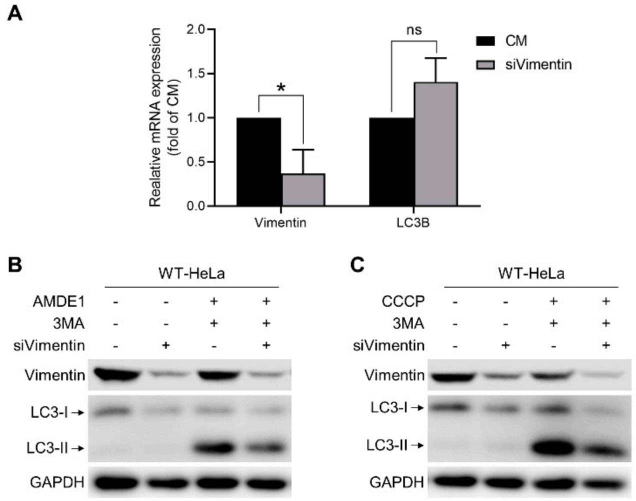

Figure S5. Vimentin was involved in Nic-induced NCLL. (A) Wild-type HeLa cells were transfected with the siRNA of *vimentin* (siVimentin) for 72 h, then the mRNA levels of Vimentin and LC3B were analyzed by qRT-PCR. (B-C) The siRNA of *vimentin*(siVimentin) was transfected to wild-type HeLa cells, followed by treatment with AMDE1 (10 μM) (B) or CCCP (30 μM) (C) in the presence or absence of 3MA (10 mM) for 6 h and then the LC3-II formation was analyzed. CM, complete medium. *p<0.05. ns, not significant.

Table S1 The LC-MS data of “molecule capture”

	**Accession**	**Description**	**Score**	**Coverage**	**# Proteins**	**# Unique Peptides**	**# Peptides**	**# PSMs**	**# AAs**	**MW [kDa]**	**calc. pI**
1	P20152	Vimentin OS=Mus musculus GN=Vim PE=1 SV=3 - [VIME_MOUSE]	1243.593	42.06	7	22	22	55	466	53.65507	5.122559
2	Q9QXS1	Plectin OS=Mus musculus GN=Plec PE=1 SV=3 - [PLEC_MOUSE]	934.4464	9.74	2	43	43	58	4691	533.8611	5.960449
3	P62806	Histone H4 OS=Mus musculus GN=Hist1h4a PE=1 SV=2 - [H4_MOUSE]	881.9098	51.46	1	6	6	43	103	11.36038	11.35596
4	Q62167	ATP-dependent RNA helicase DDX3X OS=Mus musculus GN=Ddx3x PE=1 SV=3 - [DDX3X_MOUSE]	396.5608	12.24	4	7	8	17	662	73.05603	7.181152
5	P60710	Actin, cytoplasmic 1 OS=Mus musculus GN=Actb PE=1 SV=1 - [ACTB_MOUSE]	379.2931	22.4	2	2	7	21	375	41.70973	5.478027
6	P63017	Heat shock cognate 71 kDa protein OS=Mus musculus GN=Hspa8 PE=1 SV=1 - [HSP7C_MOUSE]	362.7675	16.72	5	9	10	19	646	70.82722	5.516113
7	P68033	Actin, alpha cardiac muscle 1 OS=Mus musculus GN=Actc1 PE=1 SV=1 - [ACTC_MOUSE]	279.8592	21.75	4	2	7	20	377	41.99188	5.38916
8	P02535	Keratin, type I cytoskeletal 10 OS=Mus musculus GN=Krt10 PE=1 SV=3 - [K1C10_MOUSE]	259.5796	7.89	1	3	4	6	570	57.73506	5.109863
9	P52480	Pyruvate kinase PKM OS=Mus musculus	247.0927	19.59	2	9	9	15	531	57.80801	7.474121

		GN=Pkm PE=1 SV=4 - [KPYM_MOUSE]									
10	Q61879	Myosin-10 OS=Mus musculus GN=Myh10 PE=1 SV=2 - [MYH10_MOUSE]	233.9148	6.48	1	7	11	17	1976	228.855	5.541504
11	Q03265	ATP synthase subunit alpha, mitochondrial OS=Mus musculus GN=Atp5a1 PE=1 SV=1 - [ATPA_MOUSE]	228.1556	10.67	1	6	6	8	553	59.7156	9.187988
12	Q8VEK3	Heterogeneous nuclear ribonucleoprotein U OS=Mus musculus GN=Hnrnpu PE=1 SV=1 - [HNRPU_MOUSE]	227.0289	7.63	1	6	6	12	800	87.86273	6.239746
13	P10126	Elongation factor 1-alpha 1 OS=Mus musculus GN=Eef1a1 PE=1 SV=3 - [EF1A1_MOUSE]	185.0558	8.87	2	4	4	9	462	50.0821	9.012207
14	Q64523	Histone H2A type 2-C OS=Mus musculus GN=Hist2h2ac PE=1 SV=3 - [H2A2C_MOUSE]	157.7886	49.61	12	4	4	10	129	13.97984	10.90186
15	Q8BFZ3	Beta-actin-like protein 2 OS=Mus musculus GN=Actbl2 PE=1 SV=1 - [ACTBL_MOUSE]	155.8082	13.03	1	1	4	7	376	41.97699	5.490723
16	P67778	Prohibitin OS=Mus musculus GN=Phb PE=1 SV=1 - [PHB_MOUSE]	145.2343	19.49	1	5	5	6	272	29.8019	5.757324
17	P11499	Heat shock protein HSP 90-beta OS=Mus musculus GN=Hsp90ab1 PE=1 SV=3 - [HS90B_MOUSE]	132.3134	6.91	3	4	4	5	724	83.22912	5.033691
18	O35129	Prohibitin-2 OS=Mus musculus GN=Phb2 PE=1 SV=1 - [PHB2_MOUSE]	121.8405	14.05	1	4	4	9	299	33.27592	9.83252
19	Q9Z204	Heterogeneous nuclear ribonucleoproteins C1/C2 OS=Mus musculus GN=Hnrnpc PE=1	119.4068	13.1	1	4	4	9	313	34.36386	5.046387

		SV=1 - [HNRPC_MOUSE]									
20	P35550	rRNA 2'-O-methyltransferase fibrillarin OS=Mus musculus GN=Fbl PE=1 SV=2 - [FBRL_MOUSE]	110.5403	11.01	2	3	3	4	327	34.28568	10.24268
21	P48678	Prelamin-A/C OS=Mus musculus GN=Lmna PE=1 SV=2 - [LMNA_MOUSE]	103.6296	6.77	1	5	5	8	665	74.1927	6.976074
22	Q9JIK5	Nucleolar RNA helicase 2 OS=Mus musculus GN=Ddx21 PE=1 SV=3 - [DDX21_MOUSE]	97.63988	4.35	1	3	3	6	851	93.49324	9.114746
23	Q9R190	Metastasis-associated protein MTA2 OS=Mus musculus GN=Mta2 PE=1 SV=1 - [MTA2_MOUSE]	88.05667	3.74	3	2	2	3	668	74.98266	9.671387
24	P56959	RNA-binding protein FUS OS=Mus musculus GN=Fus PE=1 SV=1 - [FUS_MOUSE]	82.46597	2.32	1	1	1	2	518	52.64151	9.36377
25	P62827	GTP-binding nuclear protein Ran OS=Mus musculus GN=Ran PE=1 SV=3 - [RAN_MOUSE]	82.39336	10.19	2	2	2	3	216	24.40762	7.48877
26	Q6DFW4	Nucleolar protein 58 OS=Mus musculus GN=Nop58 PE=1 SV=1 - [NOP58_MOUSE]	74.82	3.36	1	1	1	1	536	60.30472	8.338379
27	Q7TMK9	Heterogeneous nuclear ribonucleoprotein Q OS=Mus musculus GN=Syncrip PE=1 SV=2 - [HNRPQ_MOUSE]	69.02728	4.98	1	3	3	4	623	69.58961	8.587402
28	Q91VR5	ATP-dependent RNA helicase DDX1 OS=Mus musculus GN=Ddx1 PE=1 SV=1 - [DDX1_MOUSE]	67.68	2.84	1	1	1	1	740	82.44777	7.210449

29	P16858	Glyceraldehyde-3-phosphate dehydrogenase OS=Mus musculus GN=Gapdh PE=1 SV=2 - [G3P_MOUSE]	67.64439	12.61	1	4	4	5	333	35.78721	8.250488
30	P68040	Guanine nucleotide-binding protein subunit beta-2-like 1 OS=Mus musculus GN=Gnb2l1 PE=1 SV=3 - [GBLP_MOUSE]	67.46084	11.67	1	3	3	3	317	35.05456	7.693848
31	O70133	ATP-dependent RNA helicase A OS=Mus musculus GN=Dhx9 PE=1 SV=2 - [DHX9_MOUSE]	66.44217	3.26	1	4	4	4	1380	149.3805	6.82959
32	Q922J9	Fatty acyl-CoA reductase 1 OS=Mus musculus GN=Far1 PE=1 SV=1 - [FACR1_MOUSE]	65.29958	3.69	1	2	2	3	515	59.39674	9.187988
33	P17095	High mobility group protein HMG-I/HMG-Y OS=Mus musculus GN=Hmga1 PE=1 SV=4 - [HMGA1_MOUSE]	62.63242	22.43	1	2	2	2	107	11.60714	10.31592
34	P19324	Serpin H1 OS=Mus musculus GN=Serpinh1 PE=1 SV=3 - [SERPH_MOUSE]	62.12914	5.52	1	2	2	3	417	46.50419	8.821777
35	Q8BTM8	Filamin-A OS=Mus musculus GN=Flna PE=1 SV=5 - [FLNA_MOUSE]	60.48438	0.87	1	2	2	2	2647	281.0459	6.036621
36	P61750	ADP-ribosylation factor 4 OS=Mus musculus GN=Arf4 PE=1 SV=2 - [ARF4_MOUSE]	59.67	10	1	1	1	1	180	20.38361	7.137207
37	P30999	Catenin delta-1 OS=Mus musculus GN=Ctnnd1 PE=1 SV=2 - [CTND1_MOUSE]	59.6	2.35	1	1	1	1	938	104.86	6.873535
38	Q3TEA8	Heterochromatin protein 1-binding protein	58.53448	3.07	1	2	2	2	554	60.82909	9.700684

		3 OS=Mus musculus GN=Hp1bp3 PE=1 SV=1 - [HP1B3_MOUSE]									
39	Q9CQW9	Interferon-induced transmembrane protein 3 OS=Mus musculus GN=Ifitm3 PE=1 SV=1 - [IFM3_MOUSE]	57.89	5.84	2	1	1	2	137	14.94459	7.400879
40	P13864	DNA (cytosine-5)-methyltransferase 1 OS=Mus musculus GN=Dnmt1 PE=1 SV=5 - [DNMT1_MOUSE]	56.46713	1.3	1	2	2	2	1620	183.0736	7.737793
41	Q69ZN7	Myoferlin OS=Mus musculus GN=Myof PE=1 SV=2 - [MYOF_MOUSE]	56.44	0.93	1	2	2	2	2048	233.1767	6.163574
42	Q8R081	Heterogeneous nuclear ribonucleoprotein L OS=Mus musculus GN=Hnrnpl PE=1 SV=2 - [HNRPL_MOUSE]	52.51229	5.12	1	3	3	3	586	63.92325	8.104004
43	Q9D0E1	Heterogeneous nuclear ribonucleoprotein M OS=Mus musculus GN=Hnrnpm PE=1 SV=3 - [HNRPM_MOUSE]	52.49439	5.49	1	3	3	4	729	77.59738	8.631348
44	Q9JKF1	Ras GTPase-activating-like protein IQGAP1 OS=Mus musculus GN=Iqgap1 PE=1 SV=2 - [IQGA1_MOUSE]	50.45	0.42	1	1	1	1	1657	188.6243	6.480957
45	Q9JIG8	PRA1 family protein 2 OS=Mus musculus GN=Praf2 PE=1 SV=1 - [PRAF2_MOUSE]	50.14987	6.18	1	1	1	2	178	19.46572	9.598145
46	Q9Z1Q9	Valine--tRNA ligase OS=Mus musculus GN=Vars PE=1 SV=1 - [SYVC_MOUSE]	48.46	1.74	1	2	2	2	1263	140.1271	7.76709
47	Q9DBG6	Dolichyl-diphosphooligosaccharide--protein glycosyltransferase subunit 2 OS=Mus musculus GN=Rpn2 PE=1 SV=1 -	46.73	1.43	1	1	1	1	631	69.02004	5.808105

		[RPN2_MOUSE]									
48	P46978	Dolichyl-diphosphooligosaccharide--protein glycosyltransferase subunit STT3A OS=Mus musculus GN=Stt3a PE=1 SV=1 - [STT3A_MOUSE]	44.87	2.7	1	1	1	1	705	80.54494	8.104004
49	Q921N6	Probable ATP-dependent RNA helicase DDX27 OS=Mus musculus GN=Ddx27 PE=1 SV=3 - [DDX27_MOUSE]	43.28	1.58	1	1	1	1	760	85.88516	9.246582
50	Q99JB2	Stomatin-like protein 2, mitochondrial OS=Mus musculus GN=Stoml2 PE=1 SV=1 - [STML2_MOUSE]	42.5	3.97	1	1	1	1	353	38.36122	8.865723
51	P02088	Hemoglobin subunit beta-1 OS=Mus musculus GN=Hbb-b1 PE=1 SV=2 - [HBB1_MOUSE]	42.02	6.8	3	1	1	1	147	15.83015	7.649902
52	Q9DBE9	pre-rRNA processing protein FTSJ3 OS=Mus musculus GN=Ftsj3 PE=1 SV=1 - [SPB1_MOUSE]	40.52	0.95	1	1	1	1	838	95.47388	8.382324
53	Q5SS00	DBF4-type zinc finger-containing protein 2 homolog OS=Mus musculus GN=Zdbf2 PE=2 SV=1 - [ZDBF2_MOUSE]	39.7617	0.28	1	1	1	4	2493	273.5778	5.224121
54	Q6NS46	Protein RRP5 homolog OS=Mus musculus GN=Pdcd11 PE=1 SV=2 - [RRP5_MOUSE]	38.4	0.48	1	1	1	1	1862	207.6486	8.748535
55	Q8BGZ7	Keratin, type II cytoskeletal 75 OS=Mus musculus GN=Krt75 PE=1 SV=1 - [K2C75_MOUSE]	37.9	1.81	3	1	1	1	551	59.70392	8.309082
56	Q8VEM8	Phosphate carrier protein, mitochondrial	37.74	2.8	1	1	1	1	357	39.60646	9.26123

		OS=Mus musculus GN=Slc25a3 PE=1 SV=1 - [MPCP_MOUSE]									
57	P11276	Fibronectin OS=Mus musculus GN=Fn1 PE=1 SV=4 - [FINC_MOUSE]	37.23	1.37	1	3	3	3	2477	272.3678	5.592285
58	Q60972	Histone-binding protein RBBP4 OS=Mus musculus GN=Rbbp4 PE=1 SV=5 - [RBBP4_MOUSE]	37.14	1.88	2	1	1	1	425	47.62607	4.894043
59	Q99JR8	SWI/SNF-related matrix-associated actin-dependent regulator of chromatin subfamily D member 2 OS=Mus musculus GN=Smarcd2 PE=1 SV=2 - [SMRD2_MOUSE]	36.12	1.88	1	1	1	1	531	59.04763	9.64209
60	P60843	Eukaryotic initiation factor 4A-I OS=Mus musculus GN=Eif4a1 PE=1 SV=1 - [IF4A1_MOUSE]	36.11225	2.46	2	1	1	2	406	46.12456	5.478027
61	Q8BX17	Gem-associated protein 5 OS=Mus musculus GN=Gemin5 PE=1 SV=2 - [GEMI5_MOUSE]	34.95	0.53	1	1	1	1	1502	166.4868	6.712402
62	P28740	Kinesin-like protein KIF2A OS=Mus musculus GN=Kif2a PE=1 SV=2 - [KIF2A_MOUSE]	34.35	1.13	1	1	1	1	705	79.70658	6.727051
63	P61211	ADP-ribosylation factor-like protein 1 OS=Mus musculus GN=Arl1 PE=1 SV=1 - [ARL1_MOUSE]	33.98	8.84	1	1	1	1	181	20.39847	5.719238
64	Q3UJB9	Enhancer of mRNA-decapping protein 4 OS=Mus musculus GN=Edc4 PE=1 SV=2 -	33.66	0.57	1	1	1	2	1406	152.3892	5.782715

		[EDC4_MOUSE]									
65	Q9CX86	Heterogeneous nuclear ribonucleoprotein A0 OS=Mus musculus GN=Hnrnpa0 PE=1 SV=1 - [ROA0_MOUSE]	33.65	2.3	4	1	1	1	305	30.51173	9.305176
66	P62315	Small nuclear ribonucleoprotein Sm D1 OS=Mus musculus GN=Snrpd1 PE=1 SV=1 - [SMD1_MOUSE]	33.16	10.92	1	1	1	1	119	13.27336	11.56104
67	Q9JJW6	Aly/REF export factor 2 OS=Mus musculus GN=Alyref2 PE=1 SV=1 - [ALRF2_MOUSE]	32.83712	5.05	2	1	1	2	218	23.71592	10.0376
68	P12382	ATP-dependent 6-phosphofructokinase, liver type OS=Mus musculus GN=Pfkl PE=1 SV=4 - [PFKAL_MOUSE]	30.34	1.15	3	1	1	1	780	85.30541	7.166504
69	Q920E5	Farnesyl pyrophosphate synthase OS=Mus musculus GN=Fdps PE=1 SV=1 - [FPPS_MOUSE]	29.94	3.68	1	1	1	1	353	40.55569	5.655762
70	Q8BKS9	Pumilio domain-containing protein KIAA0020 OS=Mus musculus GN=Kiaa0020 PE=1 SV=2 - [K0020_MOUSE]	29.24	1.24	1	1	1	2	647	72.75383	9.656738
71	Q8BND3	WD repeat-containing protein 35 OS=Mus musculus GN=Wdr35 PE=1 SV=3 - [WDR35_MOUSE]	29.06	0.59	1	1	1	1	1181	133.9055	6.341309
72	Q91VR2	ATP synthase subunit gamma, mitochondrial OS=Mus musculus GN=Atp5c1 PE=1 SV=1 - [ATPG_MOUSE]	28.88	3.36	1	1	1	1	298	32.86514	9.012207
73	Q8VDJ3	Vigilin OS=Mus musculus GN=Hdlbp PE=1 SV=1 - [VIGLN_MOUSE]	28.22	1.42	1	2	2	2	1268	141.6553	6.873535

74	Q9CQY5	Magnesium transporter protein 1 OS=Mus musculus GN=Magt1 PE=1 SV=1 - [MAGT1_MOUSE]	27.99	2.69	1	1	1	1	335	37.94441	9.759277
75	Q8CGC7	Bifunctional glutamate/proline--tRNA ligase OS=Mus musculus GN=Eprs PE=1 SV=4 - [SYEP_MOUSE]	27.91	0.53	1	1	1	1	1512	169.9716	7.664551
76	P29341	Polyadenylate-binding protein 1 OS=Mus musculus GN=Pabpc1 PE=1 SV=2 - [PABP1_MOUSE]	27.57	3.62	1	2	2	2	636	70.62587	9.495605
77	Q8BL97	Serine/arginine-rich splicing factor 7 OS=Mus musculus GN=Srsf7 PE=1 SV=1 - [SRSF7_MOUSE]	27.3	3.37	1	1	1	1	267	30.79884	11.89795
78	Q99P88	Nuclear pore complex protein Nup155 OS=Mus musculus GN=Nup155 PE=1 SV=1 - [NU155_MOUSE]	27.27	0.58	1	1	1	1	1391	155.0189	6.150879
79	Q5XJY5	Coatomer subunit delta OS=Mus musculus GN=Arcn1 PE=1 SV=2 - [COPD_MOUSE]	27.11	1.76	1	1	1	1	511	57.1932	6.214355
80	Q8BGH2	Sorting and assembly machinery component 50 homolog OS=Mus musculus GN=Samm50 PE=1 SV=1 - [SAM50_MOUSE]	26.86	2.99	1	1	1	1	469	51.83129	6.800293
81	P01942	Hemoglobin subunit alpha OS=Mus musculus GN=Hba PE=1 SV=2 - [HBA_MOUSE]	25.7	10.56	1	1	1	1	142	15.07576	8.221191
82	Q8K224	N-acetyltransferase 10 OS=Mus musculus GN=Nat10 PE=1 SV=1 - [NAT10_MOUSE]	24.64	0.78	1	1	1	1	1024	115.3456	8.32373

83	Q8K2Z4	Condensin complex subunit 1 OS=Mus musculus GN=Ncapd2 PE=1 SV=2 - [CND1_MOUSE]	24.48	0.57	1	1	1	1	1392	155.5667	6.379395
84	Q99ME9	Nucleolar GTP-binding protein 1 OS=Mus musculus GN=Gtpbp4 PE=1 SV=3 - [NOG1_MOUSE]	24.14	2.05	1	1	1	1	634	74.06587	9.524902
85	Q8CI11	Guanine nucleotide-binding protein-like 3 OS=Mus musculus GN=Gnl3 PE=1 SV=2 - [GNL3_MOUSE]	23.57	1.49	1	1	1	1	538	60.74881	9.114746
86	Q91V61	Sideroflexin-3 OS=Mus musculus GN=Sfxn3 PE=1 SV=1 - [SFXN3_MOUSE]	23.03	2.8	2	1	1	1	321	35.38352	9.510254
87	P52927	High mobility group protein HMGI-C OS=Mus musculus GN=Hmga2 PE=1 SV=1 - [HMGA2_MOUSE]	22.24	7.41	1	1	1	1	108	11.81211	10.62354
88	Q9D0D4	Probable dimethyladenosine transferase OS=Mus musculus GN=Dimt1 PE=2 SV=1 - [DIM1_MOUSE]	21.98	2.88	1	1	1	1	313	35.25203	9.993652
89	Q62159	Rho-related GTP-binding protein RhoC OS=Mus musculus GN=Rhoc PE=1 SV=2 - [RHOC_MOUSE]	21.94	4.15	2	1	1	1	193	21.99226	6.580566
90	P15092	Interferon-activable protein 204 OS=Mus musculus GN=Ifi204 PE=1 SV=2 - [IFI4_MOUSE]	21.69	1.25	1	1	1	1	640	71.6053	8.689941
91	Q9Z110	Delta-1-pyrroline-5-carboxylate synthase OS=Mus musculus GN=Aldh18a1 PE=1 SV=2 - [P5CS_MOUSE]	20.61	1.13	1	1	1	1	795	87.21187	7.547363

92	O35286	Pre-mRNA-splicing factor ATP-dependent RNA helicase DHX15 OS=Mus musculus GN=Dhx15 PE=1 SV=2 - [DHX15_MOUSE]	20.08	1.38	1	1	1	3	795	90.94921	7.459473
